# Revisiting the trajectory of medical students’ empathy, and impact of gender, specialty preferences and nationality: a systematic review

**DOI:** 10.1186/s12909-020-1964-5

**Published:** 2020-02-17

**Authors:** Freja Allerelli Andersen, Ann-Sofie Bering Johansen, Jens Søndergaard, Christina Maar Andersen, Elisabeth Assing Hvidt

**Affiliations:** 1 University of Southern Denmark, Campusvej 55, 5230 Odense M, Denmark; 2 Research Unit of General Practice, Department of Public Health, University of Southern Denmark, J.B. Winsløws Vej 9A, 5000 Odense C, Denmark; 3 Department of Psychology, University of Southern Denmark, Campusvej 55, 5230 Odense M, Denmark; 4 Department for the Study of Culture, University of Southern Denmark, Campusvej 55, 5230 Odense M, Denmark

**Keywords:** Empathy, Medical students, Systematic review

## Abstract

**Background:**

Empathy allows a physician to understand the patient’s situation and feelings and respond appropriately. Consequently, empathy gives rise to better diagnostics and clinical outcomes. This systematic review investigates the level of empathy among medical students across the number of educational years and how this level relates to gender, specialty preferences, and nationality.

**Method:**

In accordance with the Preferred Reporting Items for Systematic Reviews and Meta-Analyses (PRISMA), the authors conducted a systematic search of studies published between February 2010 and March 2019 investigating the level of empathy among medical students. The databases PubMed, EMBASE, and PsycINFO were searched. Studies employing quantitative methodologies and published in English or Scandinavian language and examining medical students exclusively were included.

**Results:**

Thirty studies were included of which 24 had a cross-sectional and 6 a longitudinal study design. In 14 studies, significantly lower levels of empathy were reported by increase in the number of educational years. The remaining 16 studies identified both higher, mixed and unchanged levels. In 18 out of 27 studies it was reported that females had higher empathy scores than males. Only three out of nine studies found an association between empathy scores and specialty preferences. Nine out of 30 studies reported a propensity towards lower mean empathy scores in non-Western compared to Western countries.

**Conclusion:**

The results revealed equivocal findings concerning how the empathy level among medical students develops among medical students across numbers of educational years and how empathy levels are associated with gender, specialty preferences, and nationality. Future research might benefit from focusing on how students’ empathy is displayed in clinical settings, e.g. in clinical encounters with patients, peers and other health professionals.

## Background

Empathy is usually categorised as either affective (emotional), cognitive or a combination of both. The essence of affective empathy is compassion and the ability to enter into other peoples’ feelings (*Einfühlung*). Cognitive empathy is described as “*the ability to understand someone’s situation without making it one’s own”* [1]. In the clinical setting and within the context of the patient-physician relationship, it is predominantly the cognitive empathy type that is valued and strived for. Mercer and Reynolds [2] define clinical empathy as the ability to a) understand the patient’s situation, perspective and feelings (and their attached meanings); b) to communicate that understanding and check its accuracy and c) to act on that understanding with the patient in a helpful (therapeutic) way. An empathic physician is able to sense the patient’s feelings while at the same time sustaining his or her professionalism [3]. Empathy has been shown to contribute substantially to building and maintaining a good patient-physician relationship [4]. Studies on empathy among general practitioners (physicians specialised in general practice) concluded that a general practitioner’s display of empathy creates a relationship built on trust, openness, and safety and that a general practitioner’s empathic attitude makes the patient feel supported and listened to [5, 6]. Consequently, patients are more likely to disclose accurate and important information about themselves resulting in better diagnostics and clinical outcomes [7–9]. Steinhausen et al. [8] found that patients who rated their physician as having “high physician empathy” using the Consultation-and-Relational-Empathy (CARE) measure had a 20-fold higher probability of a better self-reported medical treatment outcome compared to patients who rated their physician to have “low physician empathy.” Furthermore, studying patients with diabetes, Hojat et al. [9] found a strong correlation between an empathic physician (measured through the Jefferson Scale of Physician Empathy (JSPE)) and lower values of lipoprotein cholesterol (LDL) and glycosylated haemoglobin (HbA1c). Beyond clinical outcomes, empathic communication has been shown to enhance patient satisfaction, compliance and patient empowerment [10–12]. Additionally, regarding physician-related benefits of empathy, physicians who perceive themselves as being empathic experience empathy as a source of professional satisfaction and meaningfulness protecting against burn-out [5, 13, 14]. As an offshoot of the large body of research documenting the beneficial effects of physician empathy, empathy development among medical students has become a comprehensive research topic. Moreover, the association between levels of empathy among medical students and variables such as gender, nationality and/or specialty preferences has received an increased focus among researchers. Hojat et al. [15] found that medical students interested in primary care specialties had higher empathy scores than students showing interests in technology and procedure orientated specialties. Female and male physicians are furthermore shown to approach the patient-physician relationship differently [16]. For example, female physicians value psychosocial factors more than male physicians and engage to a larger extent in patient-centred and/or relationship-centred communication [17]. These varying cultural, social and psychological influences on empathy levels are also reflected in the fact that findings from studies conducted in different countries vary to a high degree [18, 19]. Several research studies using student self-report measures to measure empathy levels have documented that a significant decline in empathy occurs among medical students as their training progresses [20, 21]. Contrary to these finding, however, other studies have shown that empathy levels among medical students increase or that they are maintained [22–24]. Neumann et al. [25] published a systematic review on student empathy in 2011, concluding on the basis of 18 studies that empathy levels decline during medical education due to, mainly, an increase in student-patient contact and interaction. Colliver et al. [26] however, conducting a meta-analysis a year earlier, concluded that student empathy levels only decrease to a minimal degree if at all. Since then, more studies on the subject have been published that assumingly reflect all the new educational initiatives taking in relation to the medical curriculum that have empathy cultivation and preservation as a key goal, such as accompanying patients on medical visits making home visits, and reading medically related literature and poetry (narrative medicine) [27, 28]. Summarising the above, empathy is an important concept in health care and within educational research. However, as a consequence of many different definitions and understandings of empathy, and of different ways of measuring empathy, research in the area has also led to ambiguous results. There is therefore a need for an updated overview and review of the most recent research evidence regarding empathy among medical students.

The aim of this study was to perform a systematic review in accordance with the Preferred Reporting Items for Systematic Reviews and Meta-Analyses Guidelines (PRISMA) [29] of the literature published between February 2010 and March 2019. We sought to answer the following questions:

1. What are the empathy levels among medical students across the number of educational years?
2. How do levels of empathy relate to gender, specialty preferences, and nationality?

## Method

### Search strategy

The review was conducted according to the PRISMA guidelines [29]. AJ and FA conducted a systematic search in March 2019 informed by the research questions. Three databases were searched: PubMed, EMBASE, and PsycINFO. The following search words were used: ‘empathy’ AND ‘medical student’ AND (‘decrease’ OR ‘increase’). Additionally, synonyms, the National Library of Medicine’s Medical Subject Heading terms (MeSH) and subject headings were identified and applied (see Additional file 1). During the full-text screening, we also performed a manual search of reference sections to identify studies not found through the database searches.

### Inclusion and exclusion criteria

Inclusion criteria were the following:

- Studies published between February 2010 and March 2019
- Quantitative studies
- Studies in English or Scandinavian language
- Study population restricted to medical students

Exclusion criteria were the following:

- Qualitative studies
- Intervention studies
- Psychometric studies
- Conference abstracts
- Non-empirical texts

### Selection of data

Titles and abstracts of the studies were screened. In the case of uncertainty, full texts were read. Disagreement between reviewers (AJ and FA) regarding inclusion of the studies was settled through discussion until concordance was reached. Afterwards, AJ and FA read the full texts of the eligible studies. Together, the authors summarised and analysed the methods, results, and discussion sections of the studies. Independently, we applied methodological quality assessment tools on the different studies according to study design. Crombie’s items [30] were applied to cross-sectional studies (*n* = 24) and consist of seven items rated as “yes” (1 point), “unclear” (0.5 points) and “no” (0 points), with a maximum of 7 points. The quality of the longitudinal studies (*n* = 6) was assessed by employing a structured 33-point checklist from Tooth et al. (see Additional file 2) [31]. Possible disagreements were discussed and settled and there was inter-rater reliability.

## Results

### Included studies

**Fig. 1:**
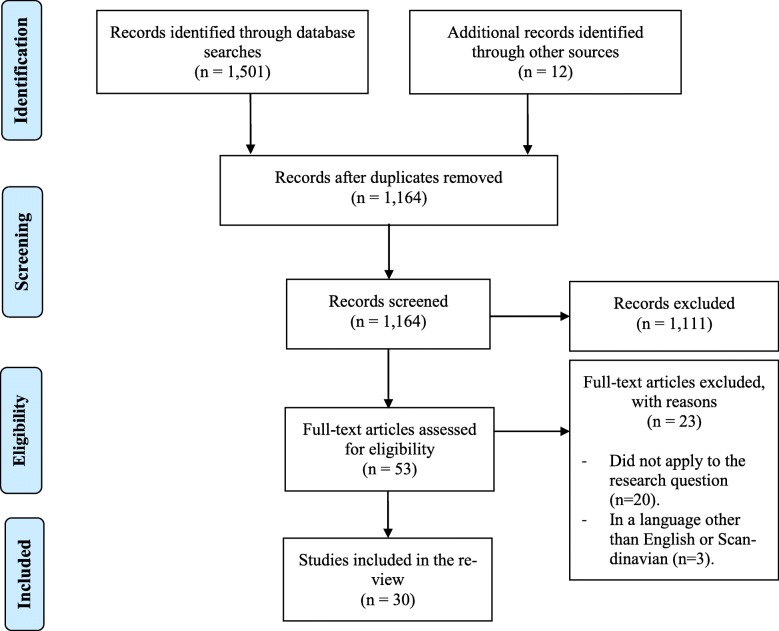
PRISMA Flowchart

The search resulted in 1501 studies, of which 347 were duplicates (see Fig. 1). A total of 1154 studies were screened by title and abstract. Among these, 41 studies were selected for full-text reading since they fulfilled the inclusion criteria. During full-text reading, reference sections were also screened, which revealed another 12 eligible studies. A total of 53 studies were full-text screened. We excluded 23 of the 53 studies since they did not apply to our aim (*n* = 20) or were in a language other than English or Scandinavian (*n* = 3). Altogether 30 studies were included in the review.

### Study characteristics

#### Study design and sample sizes

Of the 30 studies included in the review, 24 studies were cross-sectional and 6 studies longitudinal (see study characteristics and main findings in Table 1). Sample sizes of the cross-sectional studies varied from 129 [28] to 5521 [48] participants. In the longitudinal studies, sample size varied from 72 [52] to 1653 [55] participants.

**Table 1:** Results

Title	Author, year published	Country	Study objective	Study design	Sample size (response rate)	Scales^1^	Results	Comments on study quality
A latent growth model suggests that empathy of medical students does not decline over time [32]	Costa et al. 2013 [32]	Portugal	Investigate empathy throughout medical school	Longitudinal	77	JSPE-S NEO-Five-Factor Inventory	- Non-significant positive evolution in empathy scores across three time points in Portuguese undergraduate medical students that cover the preclinical/clinical transmission - A significant decline in empathy was found in females in the transition period from pre-clinical to the clinical phase of education	- Single-institution survey - Small sample size
A Quantitative Study of Empathy in Pakistani Medical Students: A Multicentered Approach [33]	Tariq et al. 2017 [33]	Pakistan	Investigate empathy among medical students in the context of patient care	Cross-sectional	1453	JSPE-S	- A statistically significant difference in JSPE scores for the 5 medical year groups - No significant difference in the JSPE scores for males and females - No significant difference in the JSPE scores across specialty preference	- Multi-center survey - Response rate unspecified
An exploration of changes in cognitive and emotional empathy among medical students in the Caribbean [34]	Youssef et al. 2014 [34]	Trinidad &Tobago	Investigate the empathy profile across all five years of training in a medical school	Cross-sectional	667 (67%)	JSPE-STEQ RMET	- Highest scores were observed when students enter medical school and the lowest scores among third-year students - Female mean scores were significantly higher than male - No significant effects of ethnicity or specialisation	- Single-institution survey - Highest response rate at year one
Characterizing changes in student empathy throughout medical school [35]	Chen et al. 2012 [35]	USA	Investigate long-term trajectories of empathy among medical students	Longitudinal	1162 (81%)	JSPE-S	- Empathy levels increased from the beginning of medical school until the end of preclinical years, followed by a decline in the third year of medical school that persisted throughout graduation - Females showed significantly higher empathy levels than males	- Single-institution survey - Varying response rate (54–99%)
Clinical empathy in medical students in India measured using the Jefferson Scale of Empathy–Student Version [36]	Chatterjee et al. 2017	India	Investigate clinical empathy and the various associated factors in medical students spanning 4 years of undergraduate study	Cross-sectional	418 (69.7%)	JSPE-S	- Mean empathy scores fell from the first to the third semester, then more or less plateaued, and then rose again in the seventh semester - Females having significantly higher empathy scores than males	- Single-institution survey
Comparative cross-sectional study of empathy among first year and final year medical students in Jimma University, Ethiopia: Steady state of the heart and opening of the eyes [37]	Dehning, S. et al. 2012	Ethiopia	Investigate whether empathy increases with medical training and identify the socio-demographic background of medical students influencing their empathy levels	Cross-sectional	237	BEES RMET	- No significant difference in emotional empathy between first-year and final year medical students - Higher cognitive empathy among final year students as compared to first-year students - Males scored lower mean cognitive and emotional empathy scores than females	- Single-institution survey - No response rate - Instruments not validated
Comparing a Self-Administered Measure of Empathy with Observed Behaviour Among Medical Students [38]	Chen et al. 2010	USA	Investigate the relationship between self-administered, JSPE-S, and observed empathy	Longitudinal	2nd year: 163 (97.6%) 3rd year: 159 (98.1%)	JSPE-SOSCE-evaluation	- Second-year students had higher JSPE-scores compared to third-year students, but the average observed empathy score for second-year students was lower than the observed empathy scores for third-year students - A trend towards a decline in measured empathy with increased clinical training with a self-administered instrument, but an improvement in observed empathy among those clinically experienced (i.e. third-year-students)	- Single-institution survey - A small number of OSCE participants
Comparison of empathy score among medical students in both basic and clinical levels [21]	Khademalhosseini et al. 2014	Iran	Investigate and compare the empathy scores of medical students between basic sciences and clinical levels	Cross-sectional	260	JSPE-S	- Empathy scores among medical students decreased by the increase in their educational years - Females had higher mean empathy students than males	- Single-institution survey - Response rate unspecified
Comparison of Empathy Skills and Conflict Tendency in Preclinical and Clinical Phase Turkish Medical Students: a Cross-Sectional Study [23]	Atay et al. 2014	Turkey	Investigate the differences and relations of empathy and conflict tendencies of medical students in preclinical and clinical phase	Cross-sectional	186 (55%)	Empathic Skill Scale B Form Conflict Tendency Scale	- A significantly higher score of empathy skills in last year medical students as compared to first-year and fourth-year students - No significant differences in empathy skill scores among males and females	- Single-institution survey - Low response rate
Cross sectional assessment of empathy among undergraduates from a medical college [39]	Shashikumar et al. 2014	India	Investigate empathy among medical students of various years	Cross-sectional	488 (75%)	JSPE-S	- Empathy declines during medical school, but only significant when comparing first-year and seventh-year - Significantly higher empathy score in females compared to males - No significant differences in empathy scores among different groups of specialty preferences	- Single-institution survey - A low number of responders in 7th semester
Decline of empathy among medical students: Dehumanization or useful coping process? [40]	Triffaux et al. 2019	Belgium	Investigate empathy decline among Belgian medical students across the different years of education and compare the level of empathy with commercial students	Cross-sectional	1353 medical students and 249 commercial students	The Basic Empathy Scale	- A significant decline in empathy scores among medical students - Significantly higher empathy scores among females in comparison with males - Significant higher empathy scores on all aspects of empathy for medical students compared to commercial students	- Single-institution survey - Unspecified response-rate
Empathic orientation among medical students from three universities in Barranquilla, Colombia and one university in the Dominican Republic [41]	Diaz Narvaez et al. 2014	Colombia The Dominican Republic	Investigate and compare empathic orientation in medical students from four universities	Cross-sectional	Universidad del Norte: *n* = 345 (40.16%) Universidad San Martín, *n* = 283 (57.4%) Universidad Libre, *n* = 695 (61.56%). Universidad Central del Este, *n* = 515 (60.38%)	JSPE-S	- Empathy values reduced as courses advanced in all schools analysed - No differences between genders	- Multi-center survey - Lack of consistency between text and table presentation of results
Empathy among undergraduate medical students: A multi-centre cross-sectional comparison of students beginning and approaching the end of their course [42]	Quince et al. 2016	United Kingdom (UK) New Zealand (NZ)	Investigate empathy among students at the beginning and end of undergraduate medical training in multiple medical schools	Cross-sectional	1139 UK: 1st/2nd year: 652 (54.9%) Final year: 487 (48.1) NZ 1st year: 721 (24%) Final year: 476 (15.2%)	JSPE-SIRI	- No significant differences in empathy scores between students starting and approaching the end of their course - Females had significantly higher empathy scores than males	- Multi-center survey - A low response rate in NZ final year
Empathy differences by gender and specialty preference in medical students: a study in Brazil [43]	Santos et al. 2016	Brazil	Investigate medical students’ empathy and to examine empathy differences by students’ socio-demographic characteristics, including gender, and specialty preference	Cross-sectional	226 (70.6%)	JSPE-S	- Consistently high scores of empathy in medical students enrolled in all levels of training - Higher empathy scores in females and students who intend to pursue people-oriented specialties	- Single-institution survey
Empathy in Chinese medical students: psychometric characteristics and differences by gender and year of medical education [44]	Wen et al. 2013	China	Investigate the psychometric properties of the JSPE-S among a sample of Chinese medical students and investigate the primary levels of empathy of the medical students and analyse group differences	Cross-sectional	753 (83.5%)	JSPE-S	- Statistically significant differences in empathy scores among medical students in different years of medical school. The first-year students had the lowest empathy scores and the fourth-year students had the highest empathy scores. - Significant higher empathy scores among females	- Single-institution survey - Applied mean data as a substitution for missing data
Empathy in Chinese eight-year medical program students: differences by school year, educational stage, and future career preference [45]	Li et al. 2018	China	Investigate the factor structure of a Chinese version of the JSPE-S with Chinese students, as well as to assess the differences in empathy scores	Cross-sectional	442 (83%)	JSPE-S	- A statistically significant difference in the mean JSPE-S scores in different school years. There was a difference between fifth- and sixth-year students (109.1 vs. 101.2). The seventh-year students had significant differences in empathy scores when compared to the first-year students (99.5 vs.107.6) and seventh-year students differed from fifth-year students (99.5 vs. 109.1) - No significant differences in empathy scores among females and males - A statistically significant difference in empathy mean score according to future career preference. Students who preferred not to become doctors had lower empathy than those who did prefer to become doctors	- Single-institution survey - Participants among the top level of medical students in China
Empathy in Iranian medical students: A comparison by age, gender, academic performance and specialty preferences [46]	Benabbas et al. 2016	Iran	Investigate self-reported empathy and its alteration during medical school in Iranian medical students	Cross-sectional	459 (91.8%)	JSPE-S	- The mean score of empathy among interns was significantly lower than both trainees and preclinical students - No statistically significant differences in empathy scores between genders - Empathy score was not related to specialty choice	- Single-institution survey
Empathy in Iranian medical students: Measurement model of the Jefferson Scale of Empathy [47]	Shariat et al. 2013	Iran	Investigate empathy in Iranian medical students, as well as to determine the measurement model and psychometric properties of JSE in Iranian students	Cross-sectional	1187 (76.7%)	JSPE-S	- Significant decreasing linear trend in the score of empathy with increasing years of education - Females outscored male students in empathy - JSPE-S showed acceptable internal consistency and re-test reliability	- Multi-institution survey - Variable response rates at different universities
Empathy in Korean medical students: Findings from a nationwide survey [48]	Park et al. 2015	Korea	Investigate empathy scores of medical students throughout the country	Cross-sectional	5521 (39%)	JSPE-S	- Lower empathy score in males, those in higher grades, and among undergraduate students	- Multi-center survey - Low response rate
Empathy in senior year and first year medical students: a cross-sectional study [27]	Magalhães et al. 2011	Portugal	Investigate the differences in empathy scores between first-year and senior students, between genders, and between specialty preferences	Cross-sectional	476 (92%)	JSPE-S	- Sixth-year students had higher empathy scores than first-year medical students - Females had higher empathy scores than males - No significant difference in empathy scores among different speciality preferences	- Single-institution survey
Empathy in UK medical students: differences by gender, medical year and specialty interest [49]	Tavakol et al. 2011	United Kingdom	Investigate the relationship between undergraduate medical students’ empathy scores	Cross-sectional	853 (68.2%)	JSPE-S	- There was no significant difference between the mean empathy scores across year groups - Females scored significantly higher empathy scores on the JSPE-S - Medical students choosing people-orientated specialties were more empathic than students choosing technology-orientated specialties	- Single-institution survey - Missing data were replaced with the mean of all values
Empathy score among medical students in Mashhad, Iran: study of the Jefferson Scale of Physician [50]	Rezayat et al. 2018	Iran	Investigate psychometric characteristics of the JSPE scale among medical students in Mashhad, Iran	Cross-sectional	640	JSPE-S	- Empathy score among medical students decreased when their educational years increased - Higher empathy scores in females than in male students - The overall rate of empathy in basic sciences period was more than in the clinical period - Empathy decreased with increasing age	- Single-Institution survey. - No response rate - Selection bias due to exclusion criteria
Erosion of empathy during medical training by gender. A cross-sectional study [51]	Calzadilla-Núñez et al. 2017	Colombia Ecuador	Investigate whether empathic erosion is a general phenomenon in the schools of medicine included in the study and its relation to gender	Cross-sectional	University 1: 278 (98%) University 2: 756 (77.86%)	JSPE-S	- There are no general patterns of how overall empathy and its components behave over school years - Males and females do not have the same empathic response	- Multi-center survey - Some educational years have small sample sizes - No assessment of statistical significance
How well do medical students rate and communicate clinical empathy? [52]	Lim et al. 2013	New Zealand	Investigate the teaching, learning and communication skills of clinical empathy in New Zealand medical students; in particular to determine if there is a similar decline during training in that country, and how well students, peers, and tutors recognise good skills and communication of empathy	Longitudinal	72	JSPE-S= BECCI	- Self-reported empathy declines during undergraduate medical training - No significant gender difference in self-reported empathy scores was observed	- Single-institution survey
Level of Empathy among Medical Students in Kuwait University [53]	Hasan et al. 2013	Kuwait	Investigate the level of empathy among medical students at various years of study and other factors	Cross-sectional	264 (56%)	JSPE-SZKPQ-50-CCPSS-10	- Increasing empathy level with the academic year, which peaked in the 4th-year, followed by a slight drop in subsequent years - A higher level of empathy among females - The desired specialty was not significantly associated with levels of empathy	- Single-institution survey - A non-validated Arabic version of empathy scales
Maintaining empathy in medical school: It is possible [24]	Hegazi et al. 2013	Australia	Investigate levels of empathy in University of Western Sydney (UWS) Medical School students across the different years of undergraduate medical education	Cross-sectional	404 (69.78%)	JSPE-S	- No significant difference in empathy scores in relation to the year of the medical course - Gender difference in levels of empathy, favouring females	- Single-institution survey.
Malaysian Medical Students’ self-reported Empathy: A cross-sectional Comparative Study [54]	Williams et al. 2015	Malaysia	Investigate empathy levels between first-year and second-year medical students	Cross-sectional	1st year: 122 (100%) 2nd year: 71 (70%)	JSPE-S	- Self-reported empathy levels declined significantly from first-year to second-year - No significant differences in empathy scores between genders	- Single-institution survey
Rethinking empathy decline: results from an OSCE [28]	Teng et al. 2017	USA	Investigate observed empathy among medical students in different clerkship years using an OSCE	Cross-sectional	129	MPCC	- Found a possible trend towards higher MPCC among students in their second clerkship year compared with students in their first-year (*p* = 0.09), which became more significant when adjusting for outliers (*p* = 0.05) - There was no difference in performance by gender. - Students who intended to pursue a “people-orientated” specialty score higher in handling the patient’s frustration	- Single-institution survey - Small sample size
Stability of empathy among undergraduate medical students: A longitudinal study at one UK medical school [55]	Quince et al. 2011	United Kingdom	Investigate the following questions: 1. Do men and women medical students differ in empathy? 2. Does empathy change amongst men and women over time?	Longitudinal	1653	IRI	- Compared to females, males recorded lower levels of affective empathy throughout their course and lower levels of cognitive empathy for part of their medical course - Male’s affective empathy declined slightly across the course overall, while female’s affective empathy showed no change. Neither male nor female showed any change in cognitive empathy during the course	- Single-institution survey
The complexity of empathy during medical school training: Evidence for positive changes [56]	Smith et al. 2017	USA	Investigate multiple facets of empathy	Longitudinal	122	JSPE-SQCAE	- JSPE-S empathy scores decreased throughout training - Students exhibited an increase in QCAE total score over time - Females exhibited higher levels of self-reported empathy	- Single-institution survey - Small sample-size

JSPE-S: Jefferson Scale of Empathy – Student Version: Measures affective empathy. The scale studies the empathic relationship between the medical student and the patient. It consists of 20 items on a 7-point Likert scale, with a minimum of 20 and a maximum of 140 points. A high score indicates a more empathic orientation [57]

NEO-Five Factor Inventory: Measures personality. It consists of 60 items that depict the following five dimensions: Neuroticism, Extraversion, Agreeableness, Openness to Experience and Consciousness. Responders answer the degree of agreement or disagreement on a 5-point Likert scale from 0 to 4 [58]

TEQ: Toronto Empathy Questionnaire: A self-reported scale. Consists of 16 questions which are designed to measure the affective component of empathy. The questions are scored on a 5-point Likert scale with a total score out of 64. A high total score accounts for a high level of emotional empathy [34]

RMET: Reading the mind in the eyes: Measures cognitive empathy by depicting the eye region in 36 photographs. The participant is required to identify the emotion being expressed and select an answer from four options. One point for each correct answer. The maximum score is 36 with mean scores typically ranging from 24 to 30 [34]

BEES: Balanced Emotional Empathy Scale: Measures affective empathy by evaluating responses to different fictive situations and life events. It consists of 30 items of which 15 are positively worded and 15 are negatively worded. Responders answer the degree of agreement or disagreement on a 9-point Likert-scale. A high total score accounts for a high level of emotional empathy [37]

OSCE: Objective structured clinical examinations [38]

Empathic Skill Scale B Form: Measures empathic skill level. It consists of six psychological problems concerning daily life and 12 types of reactions to each of these problems. Responders answer on a Likert type scale. The highest achievable score is 219 and the lowest score is 64 [23]

Conflict Tendency Scale: Measures adults’ communication skills and characterise their conflicts. It consists of 53 items - 31 positives and 22 negatives. The positive items depict a communication conflict. Negative items depict no conflict [23]

IRI: Interpersonal Reactivity Index: Consists of four scales which includes seven items - in total 28 items. The four scales consist of ‘Perspective taking’; ‘Empathic concern’; ‘Fantasy’ and ‘Personal distress’. Altogether these items embrace some of the cornerstones of empathy [59, 60]

BECCI: Behaviour Change Counselling Index: Measure of practitioner’s consultation skills about behaviour change. It consists of 11 items. An example of an item is: “Practitioner demonstrates sensitivity to talking about other issues”. Responders answer on a 5-point-Likert scale ranging from 0 to 4 [52]

ZKPQ-50-CC: Zuckermann-Kuhlman Personality Questionnaire: Measures personality types. It consists of 50 true-or-false questions that depict the following five dimensions: aggression and hostility, impulsive sensation seeking, neuroticism and anxiety, activity and sociability [61]

PSS-10: Perceived Stress Scale 10-item version: Measures the level of stress. It consists of 10 items that depict the level of stress and the handling of this. A high score indicates a high level of stress [62]

MPCC: Measure of Patient Centered Communication: Measures observed empathy. It consists of three dimensions: 1) Exploring the disease and illness experience; 2) Understanding the whole person, and; 3) Finding common ground [63]

QCAE: Questionnaire of Cognitive and Affective empathy: Measures affective and cognitive components of empathy. It consists of 31 items that depict five subscales: Perspective taking, Online Stimulation, Emotion Contagion, Proximal Responsivity, Peripheral Responsivity. Responders answer their degree of agreement or disagreement on a 4-point Likert scale [64]

#### Scales

All cross-sectional studies employed the Jefferson Scale of Physician Empathy student version (JSPE-S), except for four studies using the following scales: the Basic Empathy Scale [40], Measure of Patient-Centered Communication (MPCC) [28], Reading the Mind in the Eyes (RMET) and Balanced Emotional Empathy Scale (BEES) [37], and Empathic Skill Scale Form B and Conflict Tendency Scale [23].

All longitudinal studies used JSPE-S except for one that applied the Interpersonal Reactivity Index scale (IRI) [55]. One longitudinal study applied both an observational Objective Structured Clinical Examinations (OSCE) evaluation and JSPE-S [38]. Likewise, a cross-sectional study used the Measure of Patient-Centered Communication (MPCC), which is also an observational scale that measures empathy [28].

#### Country

The studies were conducted in 20 different countries.

The Western countries were Australia [24], Belgium [40], New Zealand [42, 52], Portugal [32], USA [28, 35, 38, 56], United Kingdom [42, 49, 55].

The non-Western countries were Brazil [43], China [44, 45], Colombia [41, 51], Dominican Republic [41], Ecuador [51], Ethiopia [37], India [36, 39], Iran [21, 46, 47, 50], Korea [48], Kuwait [53], Malaysia [54], Pakistan [33], Trinidad and Tobago [34] and Turkey [23].

### Quality assessment and risk of bias in the included studies

The quality assessment tools were used to identify the risk of bias. All included studies employed self-reporting questionnaires. Consequently, reporting bias was present which may have influenced the results. Three studies used small sample sizes, including respectively 129 [28], 77 [32], and 122 [56] study participants Hence, the findings of those studies may not be representative of the student population measured and it might over- and/or underestimate the outcome measures.

Out of 30 studies, 24 were single-institution studies [21, 23, 24, 27, 28, 32, 34–40, 43–46, 49, 50, 52–56] making the results of these studies less generalisable and hereby affecting the studies’ external validity.

One obvious limitation of the cross-sectional studies design was their inability to report changes over time. On the contrary, longitudinal studies could describe changes over time. Only one study used a control group of non-medical students, increasing its quality since it enabled comparison.

All studies, except for one [37], employed validated scales to examine the level of empathy. One study [53] employed the English validated JPSE-S on students who did not have English as their native language.

### The levels of empathy across number of educational years

Significantly lower levels of empathy by increase in number of educational years were found in 14 out of 30 studies. Of these, 12 were cross-sectional studies [21, 33, 34, 39–41, 45–48, 50, 52, 54] and two were longitudinal [52, 56]. All except one [55] of the cross-sectional studies used JSPE-S. Four cross-sectional studies [23, 27, 28, 44] reported a higher level of empathy among medical students at a higher year of medical school. Five cross-sectional studies [24, 42, 43, 49, 51] and one longitudinal study [32] found no statistically significant difference in empathy scores across the different years of medical education. Hasan et al. [53] reported higher empathy scores with higher educational years up until the fourth year, where a decreasing trend was observed. A cross-sectional study [37] differentiated between emotional and cognitive empathy and found a higher cognitive empathy level in final year students compared to first-year students. On the contrary, a longitudinal study [55] found no change in cognitive empathy.

Chen et al. [38] conducted a longitudinal study, applying both self-administered empathy measures and observed empathy in an OSCE. It showed higher self-administered empathy scores among second-year students compared to third-year students and the opposite for the observed empathy scores. In another longitudinal study by Chen et al. [35] higher levels of empathy were found up to the third-year of education, followed by a persistent decline.

Smith et al. [56] conducted a longitudinal study applying both JSPE-S and the Questionnaire of Cognitive and Affective Empathy (QCAE). The two scales revealed incongruent results: the QCAE score increased over time while JSPE-S measured a decrease over time.

### Gender

Female students were reported to have higher empathy scores compared to male students in 16 cross-sectional and 2 longitudinal studies [21, 24, 27, 34–37, 39, 40, 42–44, 47–50, 53, 56]. One longitudinal study by Quince et al. [55] found a lower level of emotional empathy among men compared to women who did not show any change. No gender differences were found in relation to cognitive empathy and no differences between genders were detected in seven cross-sectional [23, 28, 33, 41, 45, 46, 54]. Three studies did not investigate the differences in empathy across genders [32, 38, 51].

### Specialty preferences

Nine cross-sectional studies investigated a possible relation between empathy scores and specialty preferences of the students [27, 28, 33, 34, 39, 43, 45, 46, 53]. Three studies detected higher levels of empathy among students who preferred a “people-orientated” specialty [28, 43, 45]. No association between specialty preferences and empathy scores was found in the remaining six studies. None of the longitudinal studies examined specialty preferences.

### Western and non-Western countries

Out of the thirty studies, nine cross-sectional studies that all applied JSPE-S, from India [36, 39], Kuwait [53], China [44, 45], Korea [48], Iran [46, 50] and Pakistan [33], reported lower mean empathy scores compared to Western countries.

## Discussion

### Main findings

This systematic review aimed to investigate the level of empathy among medical students across the educational years and how the measured empathy levels relate to gender, specialty preferences, and nationality. In reviewing studies from 20 different countries, variations were found in the level of empathy among medical students across the number of educational years. Nearly half of the included studies [21, 33, 34, 39–41, 45–48, 50, 52, 54, 56], of which only two [52, 56] were longitudinal, reported lower empathy scores with higher educational years. The remaining 17 studies [23, 24, 27, 28, 32, 35–38, 42–44, 49, 51, 53, 55, 56] identified both higher, mixed or unchanged levels of empathy throughout the medical education.

Most studies [21, 24, 27, 34–37, 39, 40, 42–44, 47–50, 53, 55, 56] found a tendency towards higher levels of empathy among female students as compared to male students. Out of nine cross-sectional studies, only three [28, 43, 45] reported an association between empathy and specialty preferences. Furthermore, studies from non-Western countries reported a lower level of mean empathy scores as compared to Western countries. These findings thus differed from the previous review by Neumann et al. [25] which concluded that empathy decreases by an increase in the educational years particularly among those preferring “non-people-orientated” specialities. While different results might be explained by differences in study populations, study design (longitudinal vs cross-section), the instrument used, local culture, etc., this review tells us that we cannot make the often quoted statement that “empathy declines with level of training”.

### Possible explanations for lower and higher levels of empathy

In the literature, several explanations for a decline in empathy have been discussed without demonstrating a clear causal relationship. Some scholars point to the phenomenon of burnout among medical students and refer to the association found in the literature between high burnout level among medical students and low empathy score [65–67]. Related, stress among medical students [68–70] has also been shown to correlate negatively with empathy [69]. Another explanation put forward in the literature for empathy decline is increased patient contact during clinical training [34, 35, 38, 45, 46, 50, 52]. Chen et al. [38] explained the development towards lower levels of empathy during clinical training as a result of an acculturation process in which superiors and mentors try to protect their students against psychological distress by cultivating a climate of cynicism, emotional distance and detachment among medical students in their contact with patients and at the same time try to safeguard “professionalism” in the clinical setting. Moreover, Li et al. [45] stated that clinical training might encompass intense patient-physician relationships, long working hours and sleep deprivation, leading to lower levels of empathy after clinical training. Furthermore, in the literature, the so-called “hidden curriculum,” lack of role models, fear and anxiety in the meeting with the patients, and increased workload are also pointed out as possible reasons for a decline in empathy [46, 71, 72]. Another explanation mentioned in the literature is that the medical curriculum focuses more on diagnosis and treatment than humanistic values [73]. Shapiro et al. [71] also stated that the biomedical discourse has diverted the students’ focus from empathy leading him/her to adopt a mechanistic view on illness that might reduce the patients to a disease or an object.

Discussing the increases in empathy levels that were documented in some of the reviewed studies, Magalhaes et al. [27] pointed out that the medical curriculum has an increased focus on the development of empathy as the educational years progress and that students have increasingly reached an acknowledgment of the importance of empathy in the patient-physician relationship. This point of view was put forward as a possible explanation for the documented increases in empathy. Furthermore, training and competence acquirement through clinical training of communication skills have also been proposed as an explanation for the tendency towards higher levels of empathy in senior year medical students [27, 28]. In relation to these explanations it should also be kept in mind that the medical curriculum varies across countries and medical schools.

### Gender differences

In the literature, varying explanations for gender differences are suggested. Bertakis et al. [16] found that females are more receptive to emotional signals than males. Furthermore, they are said to show more interest in the patient’s family and social life, thereby being able to achieve a better understanding of the patient and reach a more empathic relation. Shashikumar et al. [39] stated that females through evolutionary gender differences are more caring and loving.

### Nationalities

Nine studies in our systematic review reported a propensity towards lower empathy scores in non-Western compared to Western countries. All of these studies applied the JSPE-S. Shariat et al. [47] stated that awareness of the cultural differences should be kept in mind when applying the JSPE-S in cultures that differ from the USA, where the JSPE-S was developed. A Japanese psychometric study of the JSPE pointed out that Japanese patients preferred their physician to be calm and unemotional, emphasising that cultural differences could indeed explain the differences in empathy scores between countries and cultures [74].

### Specialty preferences

A possible association between levels of empathy and specialty preferences was investigated in nine of the included studies [27, 28, 33, 34, 39, 43, 45, 46, 53]. Only three studies [28, 43, 45] reported an association between higher level of empathy among people preferring “people-orientated” specialities. Engaging in an empathic understanding of the patients’ feelings and life circumstances is important in all medical specialities since showing an empathic attitude towards the patient has been shown to lead to positive effects on patients’ health outcome [8, 9]. It can be argued, however, that a focus on empathy is relevant mostly within people-orientated specialities since physicians who work in these specialities are both in need of help regarding empathy preservation (helping patients) and administration (helping themselves so as to decrease the risk of stress and burn-out) [3].

### Strengths and limitations

A strength of the present systematic review is that the literature search was conducted in three databases. Furthermore, the screening of literature and selection of studies was performed by two reviewers. Moreover, we consider the implementation of a quality assessment of all included studies as a strength. This review has several limitations. Since our search words included words that presume a change e.g. “decrease” and “increase,” our search may be too narrow, and there is a risk that relevant studies have been overlooked. Additionally, possible relevant studies in languages other than English and Scandinavian were not included. Another limitation is that only quantitative studies were included. This excluded qualitative aspects that could have contributed to a more varied and profound understanding of the quantitative findings.

### Future research

Most of the included studies applied the self-administered JSPE-S and therefore did not explore the display of empathy that takes place between the patient and the medical student. Sulzer et al. [75] stated that the JSPE-S scale focuses on thoughts and not actions. Furthermore, research has shown that self-reported empathy has only a vague association with the patient-physician relationship in the clinical setting [75]. To improve the knowledge of empathy among medical students, research that includes both cognition, action, and feelings is recommended [75]. Incorporation of non-medical students as control groups is also required in order to gain more insight into whether medical students’ levels of empathy differ compared to other university students. Furthermore, future investigators should employ a variety of research designs to investigate the important role of empathy in medical education, such as mixed methods research, observational research, and qualitative research. These studies could focus - not on self-reporting – but rather on patient perceptions of empathic student/physician behaviour. Qualitative research conducted with students could also contribute to new perspectives and insights about student-perceived factors influencing the development of empathy and its expression in clinical care. Lastly, a meta-analysis is desirable since it enables the calculation of statistical significance and heterogeneity.

## Conclusions

This systematic review including thirty studies, revealed varied and inconsistent findings on the levels of empathy among medical students. Statistically lower empathy scores by an increase in educational years were found in 14 studies. The remaining studies reported higher [4] and unchanged [6] scores in empathy. In most studies, females were reported to have higher levels of empathy than males. Study participants from non-Western countries reported a tendency towards lower mean empathy scores as compared to those from Western countries. Only a few studies reported a correlation between “people-oriented” specialty preferences and empathy scores. Future research should focus on examining relational empathy in student-patient interaction using observational scales and qualitative methodologies.

## Supplementary information

**Additional file 1.** Search protocol. Overview of the search strategy employed in the following databases PubMed, Embase and PsycINFO.

**Additional file 2.** Methodological quality assessment of the included studies. Above each table the type of quality assessment tool is indicated. In the first table (with overview of the longitudinal studies) the criterions appear in the left column and for each included study there is answered “yes” or “no” to these criterions. In the second table (with overview of the cross-sectional studies) the studies appear in the left column and the criteria items in the first row of the table. The quality assessment tool here is indicated with 0, 0.5 or 1 point per item. The total score is indicated in the right column.

## Data Availability

All data generated or analysed during this study are included in this published article.

## Abbreviations

BEES: Balanced Emotional Empathy Scale
CARE: Consultation-and-Relational-Empathy
IRI: Interpersonal Reactivity Index scale
JSPE: Jefferson Scale of Physician Empathy
JSPE-S: Jefferson Scale of Physician Empathy student version
LDL: Lipoprotein cholesterol
MeSH: Medical Subject Heading terms
MPCC: Measure of Patient-Centered Communication
OSCE: Objective Structured Clinical Examinations
PRISMA: Preferred Reporting Items for Systematic Reviews and Meta-Analyses
QCAE: Questionnaire of Cognitive and Affective Empathy
RMET: Reading the Mind in the Eyes
