# Supplementary material for: Revisiting the trajectory of medical students’ empathy, and impact of gender, specialty preferences and nationality: a systematic review
Source: BMC Med Educ. 2020 Feb 17;20:52. doi: 10.1186/s12909-020-1964-5 (PMC7027232; doi:10.1186/s12909-020-1964-5)
Supplement: Supplementary file 1 — Additional file 1. Search protocol. Overview of the search strategy employed in the following databases PubMed, Embase and PsycINFO. [file 12909_2020_1964_MOESM1_ESM.docx]

**Additional File 1, Search protocol**

| Database | Search words | MeSH/subject heading | Limits | Number of hits | Search date |
| --- | --- | --- | --- | --- | --- |
| PubMed | (empathy OR compassion OR “emotional intelligence” OR empathy [mesh])  AND  (medicine OR medical education OR medical education [mesh] OR continuing medical education OR undergraduate medical education OR medical internship OR medical graduate OR medical continuing OR medical students OR medical student OR medical students [mesh] OR medical school OR medical college OR medical schools OR medical schools [mesh] OR clinical clerkship OR clinical clerkship [mesh])  AND  (decrease OR decline OR reduce OR reduction OR erosion OR increase OR fixed OR deterioration) | Empathy  Medical education  Medical students  Medical schools  Clinical clerkship | March 2010 – March 2019 | 791 | March 31^st^ 2019 |
| Embase | (empathy OR compassion OR emotional intelligence)  AND  (medicine OR medical education OR continuing medical education OR undergraduate medical education OR medical internship OR medical graduate OR medical continuing OR medical students OR medical student OR medical school OR medical college OR medical schools OR clinical clerkship OR clinical clerkship [mesh])  AND  (decrease OR decline OR reduce OR reduction OR erosion OR increase OR fixed OR deterioration) | Empathy  Medical education  Medical student  Medical school  Erosion  Deterioration | March 2010 – March 2019 | 538 | March 31^st^ 2019 |
| PsycINFO | (empathy OR compassion OR emotional intelligence)  AND  (medicine OR medical education OR continuing medical education OR undergraduate medical education OR medical internship OR medical graduate OR medical continuing OR medical students OR medical student OR medical school OR medical college OR medical schools OR clinical clerkship OR clinical clerkship [mesh])  AND  (decrease OR decline OR reduce OR reduction OR erosion OR increase OR fixed OR deterioration) | Empathy  Medical education  Medical students | March 2010 – March 2019 | 192 | March 31^st^ 2019 |
