# Supplementary material for: Revisiting the trajectory of medical students’ empathy, and impact of gender, specialty preferences and nationality: a systematic review
Source: BMC Med Educ. 2020 Feb 17;20:52. doi: 10.1186/s12909-020-1964-5 (PMC7027232; doi:10.1186/s12909-020-1964-5)
Supplement: Supplementary file 2 — Additional file 2. Methodological quality assessment of the included studies. Above each table the type of quality assessment tool is indicated. In the first table (with overview of the longitudinal studies) the criterions appear in the left column and for each included study there is answered “yes” or “no” to these criterions. In the second table (with overview of the cross-sectional studies) the studies appear in the left column and the criteria items in the first row of the table. The quality assessment tool here is indicated with 0, 0.5 or 1 point per item. The total score is indicated in the right column. [file 12909_2020_1964_MOESM2_ESM.docx]

**Additional File 2, Methodological quality assessment**

**Longitudinal studies, 33-point checklist by Tooth et al (2004)**

| Criterions | Costa, P. et al.  2013 | Chen, D. C. et al.  2012 | Chen, D. C. et al.  2010 | Lim, B.T. et al.  2013 | Quince, T. A. et al.  2011 | Smith, K. E. et al.  2017 |
| --- | --- | --- | --- | --- | --- | --- |
| 1. Are the objectives or hypotheses of the study stated? | Yes | Yes | Yes | Yes | Yes | Yes |
| 2. Is the target population defined? | Yes | Yes | Yes | Yes | Yes | Yes |
| 3. Is the sampling frame defined? | Yes | Yes | Yes | Yes | Yes | Yes |
| 4. Is the study population defined? | Yes | Yes | Yes | Yes | Yes | Yes |
| 5. Are the study setting (venues) and/or geographic location stated? | Yes | Yes | Yes | Yes | Yes | Yes |
| 6. Are the dates between which the study was conducted stated or implicit? | No | Yes | Yes | No | Yes | Yes |
| 7. Are eligibility criteria stated? | Yes | Yes | Yes | Yes | Yes | Yes |
| 8. Are issues of “selection in” to the study mentioned? | Yes | Yes | Yes | Yes | Yes | Yes |
| 9. Is the number of participants justified? | No | No | No | No | No | No |
| 10. Are numbers meeting and not meeting the eligibility criteria stated? | Yes | Yes | Yes | Yes | Yes | Yes |
| 11. For those not eligible, are the reasons why stated? | Yes | No | Yes | Yes | No | No |
| 12. Are the numbers of people who did/did not consent to participate stated? | Yes | Yes | Yes | Yes | Yes | Yes |
| 13. Are the reasons that people refused to consent stated? | Yes | No | No | No | No | No |
| 14. Were consenters compared with nonconsenters? | No | No | No | No | No | No |
| 15. Was the number of participants at the beginning of the study stated? | Yes | Yes | Yes | Yes | Yes | Yes |
| 16. Were methods of data collection stated? | Yes | Yes | Yes | Yes | Yes | Yes |
| 17. Was the reliability (repeatability) of measurement methods mentioned? | Yes | Yes | Yes | Yes | Yes | Yes |
| 18. Was the validity (against a “gold standard”) of measurement methods mentioned? | Yes | Yes | No | Yes | Yes | Yes |
| 19. Were any confounders mentioned? | Yes | Yes | Yes | Yes | Yes | Yes |
| 20. Was the number of participants at each stage/wave specified? | Yes | Yes | Yes | Yes | Yes | Yes |
| 21. Were reasons for loss to follow-up quantified? | Yes | No | No | Yes | No | No |
| 22. Was the missingness of data items at each wave mentioned? | Yes | Yes | No | Yes | Yes | Yes |
| 23. Was the type of analyses conducted stated? | Yes | Yes | Yes | Yes | Yes | Yes |
| 24. Were “longitudinal” analysis methods stated? | Yes | Yes | Yes | Yes | Yes | Yes |
| 25. Were absolute effect sizes reported? | No | No | No | No | No | No |
| 26. Were relative effect sizes reported? | No | No | No | No | No | No |
| 27. Was loss to follow-up taken into account in the analysis? | No | No | No | No | No | No |
| 28. Were confounders accounted for in analyses? | Yes | Yes | No | No | No | Yes |
| 29. Were missing data accounted for in the analyses? | Yes | Yes | Yes | Yes | Yes | Yes |
| 30. Was the impact of biases assessed qualitatively? | No | Yes | Yes | Yes | Yes | Yes |
| 31. Was the impact of biases estimated quantitatively? | No | No | No | No | No | No |
| 32. Did authors relate results back to a target population? | Yes | Yes | Yes | Yes | Yes | Yes |
| 33. Was there any other discussion of generalizability? | Yes | Yes | Yes | Yes | Yes | Yes |

**Cross-sectional studies, Crombie’s items by Zeng et al. (2015)**

| Studies (author and year published) | 1. Appropriat-eness of design to meet the aims? | 1. Adequate description of the data? | 1. Report the response rates? | 1. Adequate represent-tativeness of the sample to total | 1. Clearly stated aims and likelihood of reliable and valid measurements | 1. Assessment of statistical significance | 1. Adequate description of statistical methods | Score |
| --- | --- | --- | --- | --- | --- | --- | --- | --- |
| Tariq et al.  2017 | 1 | 1 | 0 | 0 | 1 | 1 | 1 | 5 |
| Youssef et al.  2014 | 1 | 1 | 1 | 0 | 1 | 1 | 1 | 6 |
| Chatterjee et al.  2017 | 1 | 1 | 1 | 0 | 1 | 1 | 1 | 6 |
| Dehning et al.  2012 | 1 | 1 | 1 | 0 | 0 | 1 | 1 | 5 |
| Khademalhosseini et al.  2014 | 1 | 1 | 0 | 0,5 | 1 | 1 | 1 | 5,5 |
| Atay et al.  2014 | 1 | 1 | 1 | 0 | 1 | 1 | 1 | 6 |
| Shashikumar et al.  2014 | 1 | 0,5 | 1 | 0 | 1 | 1 | 1 | 5,5 |
| Triffaux et al.  2019 | 1 | 1 | 0,5 | 0,5 | 1 | 1 | 1 | 6 |
| Diaz Narvaez et al.  2014 | 1 | 0,5 | 1 | 1 | 0 | 1 | 1 | 5,5 |
| Quince et al.  2016 | 1 | 1 | 1 | 0,5 | 1 | 1 | 1 | 6,5 |
| Santos et al.  2016 | 1 | 1 | 1 | 0 | 1 | 1 | 1 | 6 |
| Wen et al.  2013 | 1 | 0 | 1 | 0 | 1 | 1 | 1 | 5 |
| Li et al.  2018 | 1 | 1 | 1 | 0 | 1 | 1 | 1 | 6 |
| Benabbas et al.  2016 | 1 | 1 | 1 | 0 | 1 | 1 | 1 | 6 |
| Shariat et al.  2013 | 1 | 0,5 | 1 | 1 | 1 | 1 | 1 | 6,5 |
| Park et al.  2015 | 1 | 1 | 1 | 1 | 0 | 1 | 1 | 6 |
| Magalhães et al.  2011 | 1 | 1 | 1 | 0 | 1 | 1 | 1 | 6 |
| Tavakol et al.  2011 | 1 | 1 | 1 | 0 | 0 | 1 | 1 | 5 |
| Rezayat et al.  2018 | 1 | 0 | 0,5 | 0 | 1 | 1 | 1 | 4,5 |
| Calzadilla-Núñez et al.  2017 | 1 | 1 | 1 | 0 | 1 | 0 | 1 | 5 |
| Hasan et al.  2013 | 1 | 1 | 1 | 1 | 0 | 1 | 1 | 6 |
| Hegazi et al.  2013 | 1 | 1 | 1 | 0 | 1 | 1 | 1 | 6 |
| Williams et al.  2015 | 1 | 1 | 1 | 0 | 1 | 1 | 1 | 6 |
| Teng et al.  2017 | 1 | 1 | 1 | 0 | 1 | 1 | 1 | 6 |
